# Sustainable CuIn Electrodeposition from Deep Eutectic Solvents: Influence of Ethylene Glycol and Urea

**DOI:** 10.1021/acsomega.6c02001

**Published:** 2026-07-31

**Authors:** David A. de Assis, José W. A. Crisóstomo, Ana A. C. Alcanfor, Othon S. Campos, Luciana Vieira, Filipe X. Feitosa, Hosiberto B. de Sant’Ana, Diego F. Dias, Edipo S. de Oliveira, Hamilton F. G. de Abreu, Natalia G. Sousa, Francisco M. T. de Luna, Pedro de Lima-Neto, Adriana N. Correia

**Affiliations:** † Departamento de Química Analítica e Físico-Química, Centro de Ciências, 28121 Universidade Federal do Ceará, Campus do Pici, Bl 940, 60020-181 Fortaleza, Ceará, Brazil; ‡ Departamento de Química e Física, Centro de Ciências Exatas, Naturais e da Saúde, 28126 Universidade Federal do Espírito Santo, Campus Alegre, 29500-000 Alegre, Espírito Santo, Brazil; § 28453 Fraunhofer Institute for Interfacial Engineering and Biotechnology IGB Bio-, Electro- and Chemocatalysis BioCat, Straubing Branch, Schulgasse 11a, Straubing 94315, Germany; ∥ Departamento de Engenharia Química, Centro de Tecnologia, Universidade Federal do Ceará, Campus do Pici, Bl 709, 60020-181 Fortaleza, Ceará, Brazil; ⊥ Programa de Pós-Graduação em Física, Centro de Ciências, Universidade Federal do Ceará, Campus do Pici, 60020-181 Fortaleza, Ceará, Brazil; # Departamento de Engenharia Metalúrgica e de Materiais, Universidade Federal do Ceará, Campus do Pici, 60020-181 Fortaleza, Ceará, Brazil

## Abstract

The electrodeposition of CuIn coatings on platinum was investigated using CuCl_2_.2H_2_O and InCl_3_ in the deep eutectic solvents (DES) 1ChCl:2EG (1 choline chloride: 2 ethylene glycol) and 1ChCl:2U (1 choline chloride: 2 urea). The cyclic voltammograms of the CuIn solution showed three reduction processes: the first two associated with Cu^2+^/Cu^+^ and Cu^+^/Cu and the third with In^3+^/In. Physicochemical properties, such as viscosity and specific mass, were measured at different temperatures for Cu^2+^ and In^3+^ cations. Increasing the temperature reduced viscosity and increased diffusion coefficient (D) values for the species studied in both solvents. The solvadynamic radius values decreased with increasing temperature for both species in 1ChCl:2EG, whereas the opposite was observed in 1ChCl:2U. Scanning electron microscopy (SEM) analysis of the coatings was performed potentiostatically at different temperatures and cation ratios. For 1ChCl:2EG, granular morphologies were observed, and the grain size increased with increasing temperature. By altering the In^3+^ concentration in the solution, an increase of approximately 20 wt % in the coatings was achieved. This was followed in relation to the result obtained from the equimolar solution. 1ChCl:2U coatings exhibited a granular morphology with Cu at 100 wt % at 296 K, whereas at 343 K, at more negative potentials, they exhibited 45 wt %. By altering the cation ratio, the cation with a higher concentration in the electrolytic solution predominated. The results show that examining CuIn electrodeposition in different media is essential to producing high-quality coatings.

## Graphical abstract

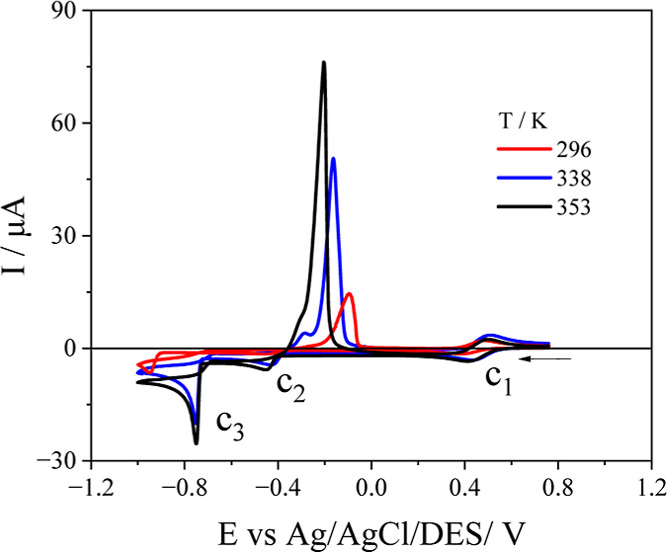

## 1 Introduction

Over the past decades, the application of II–VI semiconductor materials in the high-tech industry has been studied. They are crucial for the manufacture of laser sources, radiation detectors, electroluminescent displays, light-emitting diodes, photocatalysts, photovoltaic devices, optoelectronic devices, magnetic devices, and thermoelectric devices.^1–3^ Among these materials, metal alloys of copper (Cu) and indium (In) have gained prominence in photovoltaic applications, especially for the manufacture of thin-film solar panels.^4–9^ Among those alloys, the CuInSe_2_ (CIS), Cu­(InGa)­Se_2_ (CIGS), and Cu­(InGa)­(SeS)_2_ (CIGSSe) alloys are featured in the literature.^4,6,7,10,11^ Recently, thin-film solar cell technology achieved the highest efficiency for photovoltaic energy conversion in lab-made devices based on the CIGSSe alloy, yielding 23.35% efficiency.^12^ Another application of Cu–In alloys is as electrocatalysts for CO_2_ reduction, where Cu_11_In_9_ and Cu_2_In alloys can convert CO_2_ to CO, yielding approximately 87–95% faradaic efficiency.^13,14,16^ Other conversion products, such as H_2_, CH_3_CH_2_OH, and HCOOH, can be obtained by tuning Cu–In alloy composition to favor a determined catalyst morphology.^3,15,17^

Vacuum deposition technologies are widely used for the growth of semiconductor alloys.^5,6,10,12,18^ However, such techniques are very complex, and their high-cost processes for industrial manufacturing make them infeasible.^6^ Among many alternative methods, electrodeposition is a viable approach for preparing various advanced materials because of its ease of handling and low-cost procedures, offering advantages over other commonly used techniques.^19,20^ However, electrodeposition may be limited if applied in an aqueous medium, such as pH-dependent reactions, such as hydrogen evolution reaction (HER) and oxygen evolution reaction, which makes it challenging to electrodeposit elements with reduction potentials near those reactions, such as Ga and In, reducing their efficiency and the quality of the deposited coating.^10,20,21^

In recent years, many nonaqueous solvents have been developed to overcome the limitations of aqueous solutions. Room temperature ionic liquids (RTILs) are a class of solvents consisting of a combination of salts with a melting point below 100 °C.^22^ RTILs exhibit thermostability, high ionic conductivity, strong salt solvation, low vapor pressure, and a wide electrochemical window.^22,23^ However, one disadvantage of using ionic liquids is their hygroscopicity and their instability in the presence of water. This condition may be minimized by using RTILs with tetrafluoroborate and acetate as anions. However, some studies report that exposure to moisture alters their chemical and physical properties, releasing HF as water content increases, posing an environmental problem for industries and laboratories. Another disadvantage of RTILs is the complexity of their synthesis, which hinders their use as an alternative electrolyte.^22,24^

An alternative to RTILs is deep eutectic solvents (DES). Sharing the same qualities as RTILs, such as low vapor pressure, thermostability, wide electrochemical range, and high solvation capacity,^25,26^ DES overcome several limitations of aqueous solutions; for example, relative humidity tolerance, relative ease of preparation, and the reagents for their synthesis are economically accessible, and they are generally chemically inert.^24,27^ As a eutectic, one of its main characteristics is its low melting point, which is considerably lower than those of its pure constituents.^24,27^ The literature reports the successful electrodeposition of Cu and In from the DES electrolytes.

Considering DES based on choline chloride (ChCl) and ethylene glycol (EG) in a molar ratio of 1:2, Ghosh and Roy^28^ managed to electrodeposit Cu on a carbon steel electrode at 296 K. Vukmirovic, Adzic, and Akolkar^29^ studied the electrodeposition of Cu on platinum and glassy carbon substrates, among others. Alcanfor et al.^30^ electrodeposited In onto a copper electrode, thereby forming a Cu–In metallic phase. Bohlen et al.^31^ studied the application of the obtained In coatings for CO_2_ reduction on a Cu substrate. Considering ChCl and urea (U), there have been few studies on the electrodeposition of Cu and In. Malaquias et al.^32^ studied the electrodeposition of CuIn alloys from a choline chloride-urea-based DES (1:2 molar ratio) on a molybdenum electrode at 333 K, focusing on photovoltaic applications, underscoring the need for further research in this area.

## 2 Experimental Section

1ChCl:2EG was prepared with choline chloride (ChCl, HOC_2_H_4_N­(CH_3_)_3_Cl, Sigma-Aldrich, ≥98%) and EG (HOCH_2_CH_2_OH, Sigma-Aldrich, ≥99.8%). 1ChCl:2U solvent was prepared with choline chloride (ChCl, HOC_2_H_4_N­(CH_3_)_3_Cl, Sigma-Aldrich, ≥98%) and urea (U, NH_2_CONH_2_, Sigma-Aldrich). For the DES, the reagents were mixed at molar ratios of 1:2 (1ChCl:2EG, and 1ChCl:2U), respectively, and heated to 343 K until a homogeneous, colorless liquid formed. The electrochemical behavior of the DES in 1ChCl:2EG and 1ChCl:2U was studied. In 1ChCl:2EG: CuIn alloy and individual metals, equimolar solutions (0.050 mol L^–1^) of CuCl_2_.2H_2_O (Merck, ≥99%) and InCl_3_ (Sigma-Aldrich, ≥98%) were prepared for CuIn alloy electrodeposition, and individual metal solutions at the same concentration were also prepared. Measurements were made at 296, 338, and 353 K, and cyclic voltammetry was conducted at a scan rate of 25 mV s^–1^. The selected deposition potentials were −0.95, −0.75, and −0.75 V, respectively. Before each measurement, the working electrodes were sanded in a 600-grit SiC sanding sheet, rinsed with Milli-Q water, and dried with airflow. CuIn deposits were obtained by potentiostatic control on 20 mm^2^ rectangular platinum electrodes for 900 s for SEM analysis. Individual solutions at 0.050 mol L^–1^ were used to determine the diffusion coefficients (D) of the species Cu^2+^ and In^3+^. The experiments were performed using chronoamperometry, applying 0.0 V to the solution containing Cu^2+^ and −0.90 V to the solution containing In^3+^, for 60 s at all working temperatures. The values of the diffusion coefficients were obtained by applying Cottrell eq 1 ^33^

1 $$I\left(t\right)=\frac{nFAD_{o}^{1/2}C}{{\left(\pi t\right)}^{1/2}}$$

where n is the number of electrons transferred in the electrochemical reaction, F is the Faraday constant (96,485C mol^–1^), A is the geometric area of the working electrode (cm^2^), c is the bulk concentration of electroactive species (mol L^–1^), and t is the time (s). The values obtained for the diffusion coefficient of the Cu^2+^ and In^3+^ ions are shown in Table S1. 1ChCl:2U: CuIn alloy and individual metals and equimolar solutions (0.025 mol L^–1^) of CuCl_2_ · 2H_2_O (Merck, ≥99%) and InCl_3_ (Sigma-Aldrich, ≥98%) were prepared for CuIn alloy electrodeposition, and individual metal solutions at the same concentration were also prepared. The investigations were carried out by cyclic voltammetry at a scan rate of 25 mV s^–1^ and at 296, 338, and 353 K. The procedure used to form the electrodeposits was chronoamperometry at potentials of −1.0 V and −0.8 V for 3600 s for all Cu^2+^:In^3+^ ratios. To determine the water content of the prepared DES solutions, a Karl Fischer titrator (Metrohm-Eco Chemie) was used. Dynamic viscosity and density measurements were taken at 296, 318, 338, and 353 K using an Anton Paar Stabinger viscometer model SVM 3000. All weightings were performed using an analytical scale model TB-215D (Denver Instrument) with an accuracy of 0.01 mg. For DES preparation and prior heating of the solutions during electrochemical measurements, a heating plate model AM-10 (BIOMIXER) and/or ultrathermostatic bath (CIENLAB) were used. To assist in homogenizing the solutions, the ultrasonic bath model 03350 (QUIMIS) was used. The water used to clean the electrodes and wash the glassware was purified by the Milli-Q system (Millipore, 18.2 MΩ cm). Electrochemical experiments were performed using a model PGSTAT 30, 101, or 128N potentiostat/galvanostat (Autolab, Eco Chemie), all connected to computers via NOVA version 2.1. All electrochemical measurements were conducted in a Teflon-capped glass electrochemical cell with a conventional three-electrode arrangement. For cyclic voltammetry and chronoamperometry measurements, a lab-made, disc-shaped platinum electrode with a geometric area of 0.2 mm^2^ (99.5%, Heraeus Vectra of Brazil Ltd.a), a 45.7 mm^2^ platinum sheet (99.5%, Heraeus Vectra of Brazil Ltd.a.), and Ag/AgCl/DES were used as working, auxiliary, and pseudoreference electrodes, respectively. All potentials were calculated relative to a pseudoreference Ag/AgCl/DES electrode. The morphology of the electrodeposits was observed using a high-resolution field-emission gun scanning electron microscope (SEM) Quanta 450 FEG (FEI) equipped with an energy-dispersive X-ray spectrometer (INCA X-MAX, Oxford Instruments). For 1ChCl:2EG, X-ray diffraction (XRD) analysis of Cu–In electrodeposits was performed on a PANalytical diffractometer model Xpert-PRO MPD with Co_Kα_(λ = 1.788Å) over a measurement range of 30 to 100° using a 0.01° min^–1^ step at room temperature. The crystalline phases were analyzed using the program Panalytical Xpert Highscore Plus software version 3.0.1, with the PDF-2–2004 database. For 1ChCl:2U, sample characterization by XRD was conducted using a RIGAKU SmartLab SE automated multipurpose X-ray diffractometer with Co–Kα radiation (Kα = 1.788 Å) from a cobalt tube, operated at a voltage of 40.0 kV and a current of 35.0 mA, in a grazing angle configuration, with measurements taken at intervals of 25° to 100°, at a step of 0.04°/min. Measurements were taken at room temperature. Crystalline phases were confirmed using Panalytical XpertHighscore Plus software version 3.0d and the PDF-2–2004 database.

## 3 Results and Discussion

### 3.1 CuIn Electrodeposits in 1ChCl:2EG

The water content analysis was performed immediately after preparation of the DES and the 0.050 mol L^–1^ CuCl_2_.2H_2_O, 0.050 mol L^–1^ InCl_3,_ and 0.050 mol L^–1^ CuCl_2_.2H_2_O + 0.050 mol L^–1^ InCl_3_ in 1ChCl:2EG. The water content in the DES was 29.04 ± 0.4 ppm. For solutions containing metallic salts, the water content decreased. This value does not represent 1% of the solution volume and does not significantly alter any solution property, as demonstrated by Bezerra-Neto et al.^34^

The effect of temperature on the specific mass (ρ) and dynamic viscosity (η), as well as the water content values of the solvents before and after adding the cations of the solutions, is listed in Table S1. As the temperature increased, the specific mass of the solutions decreased, with a maximum standard deviation of 2.9% across all solutions analyzed. Regarding dynamic viscosity, temperature had a significant effect. For the 0.050 mol L^–1^ CuCl_2_.2H_2_O + 0.050 mol L^–1^ InCl_3_ solution in 1ChCl:2EG, a decrease of 83% was observed. These density and viscosity values are higher than those reported in the literature for the 1ChCl:2EG solution.^24,35^ However, this increase is due to the presence of metallic salts in the solution, since, in a constant-volume system, density is directly proportional to the total mass of the solution.

#### 3.1.1 Electrochemical Studies

The cyclic voltammograms for a 0.050 mol L^–1^ CuCl_2_.2H_2_O + 0.050 mol L^–1^ InCl_3_ solution in 1:2 ChCl: EG at different temperatures are plotted in Figure 1. The Cu^2+^/Cu^+^(c_1_) and Cu^+^/Cu^0^(c_2_) processes were observed and are associated with those shown in the Supporting Information (Figure S1). The voltammograms obtained for 0.050 mol L^–1^ CuCl_2_.2H_2_O and (B) 0.050 mol L^–1^ InCl_3_ solutions in 1ChCl:2EG using a platinum electrode between 0.76 V and −1.0 V at 25 mV s^–1^ are shown in Figure S1A. The c’_1_ process, near 0.4 V, is related to the reduction of the [CuCl_4_]^2–^ complex according to eq 2 ^29,36,37^

2 $${\left[{CuCl}_{4}\right]}^{2-}+e^{-}\to {\left[{CuCl}_{2}\right]}^{2-}+{2Cl}^{-}$$

**Figure 1:**
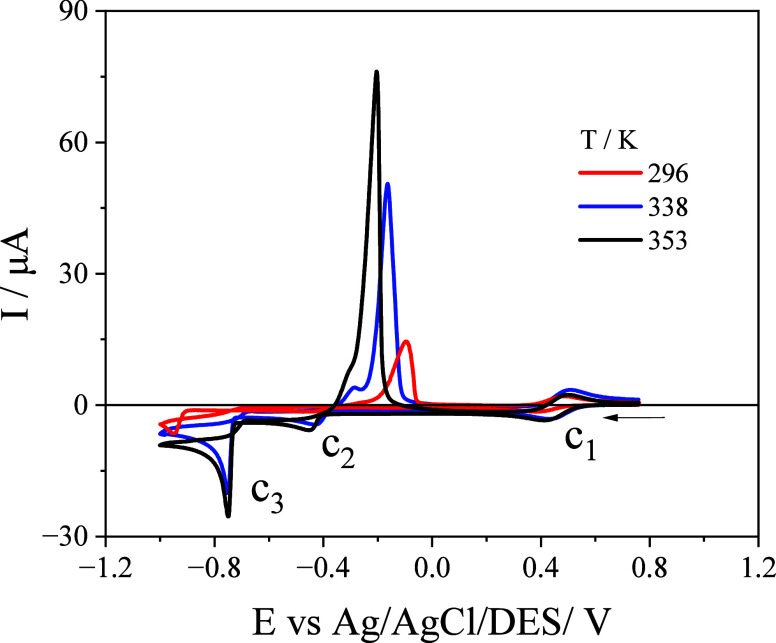
Cyclic voltammograms for 0.050 mol L^–1^ CuCl_2_.2H_2_O + 0.050 mol L^–1^ InCl_3_ in 1ChCl:2EG on a platinum working electrode at 25 mV s^–1^ at different temperatures.

The second c’_2_ process was observed at approximately −0.5 V and was related to the reduction of [CuCl_3_]^2–^ or [CuCl_2_]^−^ complexes.^28^ These potential values were not significantly altered with increasing temperature, with significant variation observed in the current values at c_1_ (three times higher at 353 K) and c’_2_, which showed values approximately 5 times higher than those obtained at 296 K. The voltammograms were obtained for a solution containing only In^3+^. At 296 K, no reduction of In^3+^/In^0^ was observed, as shown in Figure S1B. For the other temperatures, a cathodic c’_3_ process was observed at – 0.8 V, corresponding to the reduction of In^3+^, indicating that the previously observed decrease in viscosity (Table S1) facilitated the reduction of In^3+^ to elemental In, as reported by Alcanfor et al.^30^ The proposed reaction for the In^3+^ reduction process was suggested by Barrado et al.^38^ and is shown in eq 3

3 $${\left[{InCl}_{p}\right]}^{3-p}+{3e}^{-}\to {In}_{\left(s\right)}+{pCl}^{-}$$

where p represents the number of coordinated chloride ions, and the main problems proposed in this system are [InCl_4_]^−^, [InCl_5_]^2–^, and [InCl_6_]^3–^.^39^ Figure 1, as mentioned earlier, contains both cations, and a significant change was observed in the c_3_ process. This was completely affected by the presence of Cu^2+^ ions in the solution, altering the potential from −0.95 V at 296 K to values close to −0.75 V for the other temperatures, facilitating the reduction of In^3+^ by reducing it by about 0.2 V. This behavior was consistent with that observed by Alcanfor et al.^30^ in the study of In electrodeposition using a copper disc as a working electrode in 1ChCl:2EG.

Using the Cottrell equation (eq 1), the values of the diffusion coefficient (D) for both cations were obtained. The results are presented in Table S1, and for the species involved, the increase in D values was directly proportional to the rise in temperature. The values of D_Cu_^2+^ were 1.19, 3.12, 7.09, and 10.03 × 10^–7^ cm^2^ s^–1^ for 296, 318, 338, and 353 K, respectively. For In^3+^ species, values of 0.77, 2.25, 4.51, and 7.66 × 10^–7^ cm^2^ s^–1^ were obtained for 296, 318, 338, and 353 K, respectively. The D_Cu_^2+^/D_In_^3+^ ratio was between 1.3 and 1.6 times, meaning that D_Cu_^2+^ values were higher than D_In_^3+^ values at all temperatures evaluated.

To estimate the apparent activation energy (*E*_a_) of the cationic species, an Arrhenius-type plot was fitted, relating the measured D values to the inverse viscosity (η). The results obtained for Cu^2+^ and In^3+^ in 1ChCl:2EG are shown in Figure S2. The *E*_a_ values were 33.5 and 35.1 kJ mol^–1^ for Cu^2+^ and In^3+^, respectively. The solvadynamic radius (r) can be related to viscosity and D values through the Stokes–Einstein eq 4 ^40^

4 $$D_{o}=\frac{k_{B}T}{6\pi \eta r}$$

where kB is the Boltzmann constant (1.38 × 10^–23^ m^2^ kg s^–2^ K^–1^) and T is the temperature (K).

The r values for the assessed temperatures are shown in Table S1. For Cu^2+^ complexes, the solvadynamic radius decreased from 0.408 to 0.309 nm when the temperature increased from 296 to 338 K, respectively. However, at 353 K, the solvadynamic radius increased to 0.325 nm. These differences suggest that the coordination number of Cl^–^ ions complexed with Cu^2+^ ions increases as the temperature increases, altering the solvation layer and, consequently, the r value.^41^ A similar behavior may be proposed for In^3+^ ions, whose solvadynamic radius was reduced from 0.600 to 0.415 nm for the individual In^3+^ solution when the temperature increased from 296 to 353 K. Sousa et al.^42^ observed that with temperature changes, the replacement of oxygen molecules from EG by Cl^–^ ions occurred. The factors that may have influenced these results may be attributed to the relaxation of the hydrogen-bond network, which is essentially controlled by molecular interactions.^43^ The value of r depends on the temperature;^44^ that is, changes in results occur from the correlation between the central ion and the surrounding layers.^45^

### 3.2 SEM/XRD Analysis

Scanning electron microscopy (SEM) images were recorded for the CuIn coatings, as shown in Figure 2. The morphology of the coatings obtained under all electrodeposition conditions varied from a compact layer with regular grains (Figure 2A) to irregularly shaped grains (Figure 2C).

**Figure 2:**
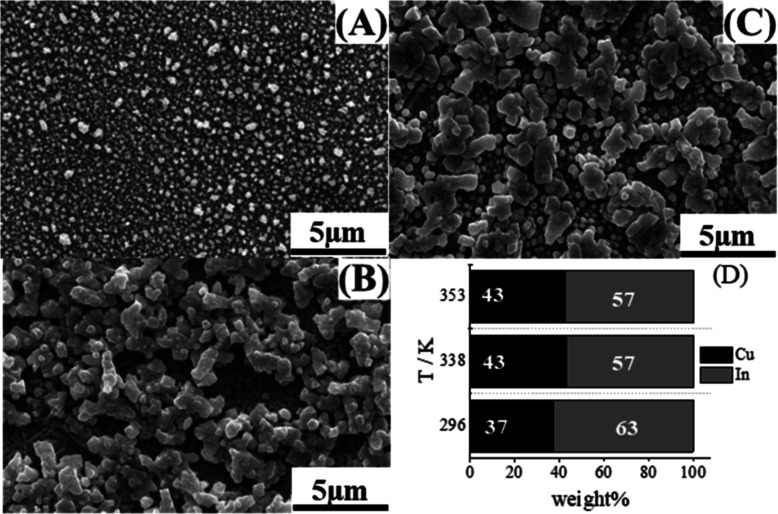
SEM images of CuIn electrodeposits over platinum for 0.050 mol L^–1^ CuCl_2_.2H_2_O + 0.050 mol L^–1^ InCl_3_ in 1ChCl:2EG solution, applying the following conditions: (A) 296 K and −0.95 V, (B) 338 K, and (C) 353 K at – 0.75 V for 900 s; (D) the percentage elemental composition of electrodeposits obtained by EDS for the coatings.

The energy-dispersive X-ray spectrometry (EDS) results (Figure 2D) showed that increasing the temperature decreased the In content in the coatings from 62.7% at 296 K to 57.5% at 353 K. The results showed that the coatings had different grain sizes, characteristic of coatings rich in In, as reported by Alcanfor et al.^30^ and Bohlen et al.^31^

XRD analysis was performed on the samples shown in Figure 2; however, the electrodeposited samples after 3600 s, to improve the signal-to-noise ratio, are shown in Figure 3.

**Figure 3:**
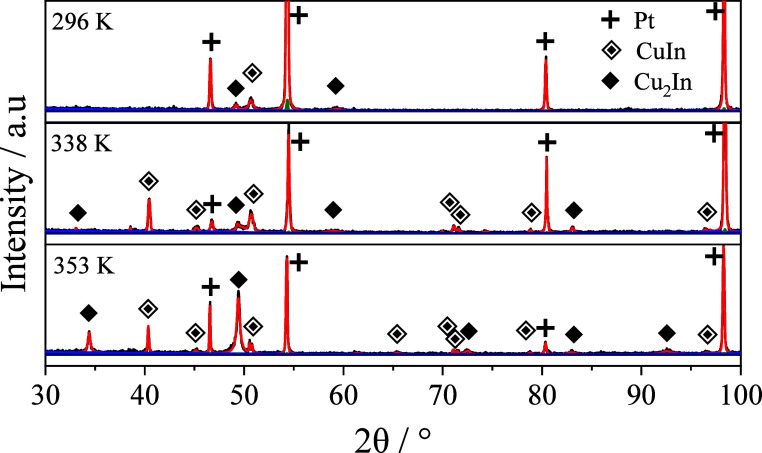
Electrodeposited Cu–In alloy diffractogram over the platinum surface for solution of 0.050 mol L^–1^ CuCl_2_ · 2H_2_O + 0.050 mol L^–1^ InCl_3_ in 1ChCl:2EG, applying the electrodeposition conditions shown in Figure 6 for 3600 s.

The deposit obtained at temperatures above 338 K exhibited two distinct crystalline alloy phases, in addition to the platinum substrate (4 diffraction peaks observed at 2θ = 46.5°, 54.3°, 80.3°, and 98.3°). CuIn alloy, space group *P*21/*m* (PDF#00–035–1150), with main reflexes at 2θ = 40.3°(200), 44.8°(102), 50.4°(−134), 70.9°(−365), 78.8°(−506), and 96.6°(−195). Another phase detect was Cu_2_In, space group *P*63/mmc (PDF#03–065–0704), located at 2θ = 34.3°(101), 49.4°(102), 72.3°(202), 82.4°(211), and 92.5°(212). As shown in Figure 3, temperature had a positive influence on the copper indium phase, whose intensity increased with temperature and brought the Cu_2_In phase above 338 K.

### 3.3 Effect on the Molar Ratio in Coatings at 296, 338, and 353 K

To evaluate the effect of In^3+^ concentration in the electrolytic bath, it was decided to assess the influence of doubling the In^3+^ concentration while maintaining the same Cu^2+^ concentration. The SEM results for 0.050 mol L^–1^ CuCl_2_.2H_2_O + 0.100 mol L^–1^ InCl_3_ in 1ChCl:2EG at different temperatures are shown in Figure 4.

**Figure 4:**
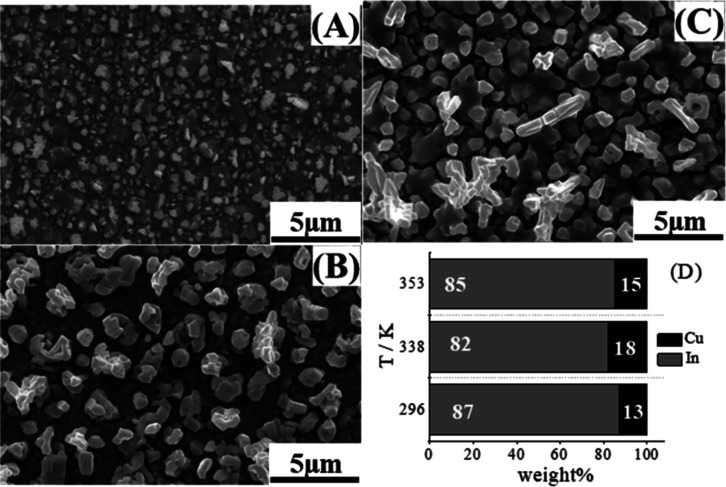
SEM images of CuIn electrodeposits over platinum for 0.050 mol L^–1^ CuCl_2_ 2H_2_O + 0.100 mol L^–1^ InCl_3_ in 1ChCl:2EG solution, applying the following conditions: (A) 296 K and −0.95 V and (B) 338 K and (C) 353 K at – 0.75 V for 900 s; (D) the percentage elemental composition of electrodeposits obtained by EDS for the coatings.

When the In^3+^ concentration was doubled, the In content in the coatings obtained increased, reaching a maximum of 87% at 296 K. Even when altering the In^3+^ proportion, the behavior was not as expected: an increase in In was not directly proportional to the temperature. Since viscosity is inversely proportional to the diffusion coefficient, differences in species transport may lead to distinct morphologies.^46^ As a result, a morphology different from that shown in Figure 4C is shown in Figure 4A. Although the In content did not change significantly, a morphological alteration occurred with a modification in the grain size of the formed overlayer. At 353 K, the beginning of the rod formation occurred. Londhe et al.^46^ reported a similar morphology when EG was used as the electrolyte to obtain CuIn coatings. Unlike the present work, they received identical morphological results with the metal content inverted. As the potential became more negative, the Cu content decreased, and the In content increased, with the Cu content always prevailing, reaching a minimum of approximately 60% at 403 K. SEM images obtained in 1:1 and 1:2 ratios in 1ChCl:2EG were subjected to grain size analysis using the methodology described in the SM. Considering the low particle count and the size behavior in the SEM images, such as particle aggregation, and the limitations of the ECD method, which does not correctly measure fused molten grains, we can draw some conclusions based on the experimental data. Table 1 presents the ECD grain-size estimates for Figures 2 and 4. The histograms for these measurements are shown in Figures S3 and S4.

**Table 1:** Grain Sizes Processed From SEM Images using the ECD Method

Cu/In (molar) ratio	temperature (K)	–E_DEP_ (V)	mean ECD ±sd (μm)
1:1	296	0.95	0.18 ± 0.15
	338	0.95	0.33 ± 0.27
	353	0.75	0.34 ± 0.27
1:2	296	0.95	0.42 ± 0.26
	338	0.95	0.60 ± 1.03
	353	0.75	0.60 ± 0.62

As shown in Figures 2 and 4, the morphology of the CuIn electrodeposits in 1ChCl:2EG depended on the molar ratio; the molar ratio directly affected the main ECD in the electrodeposited coating. The average ECD value almost doubled when the InCl_3_ concentration also doubled, indicating that the In in the CuIn alloys increased the grain size of the electrodeposits, considering the same temperature in both experiments. On the other hand, the total In composition in the electrodeposit also doubled when the InCl_3_ concentration doubled, indicating that the In content in CuIn increased with the solution concentration.

### 3.4 CuIn Electrodeposits in 1ChCl:2U

The water content analysis was performed immediately after preparing 0.025 mol L^–1^ CuCl_2_ 2H_2_O, 0.025 mol L^–1^ InCl_3_, and 0.025 mol L^–1^ CuCl_2_ 2H_2_O + 0.025 mol L^–1^ InCl_3_ in 1ChCl:2U. The water concentration in the solution with the two cations was 30.6 ppm. The effect of temperature on the specific mass (ρ), dynamic viscosity (η), and water content of the solutions is listed in Table S2.

### 3.5 Electrochemical Studies

As the temperature increases, all specified parameters decrease. The cyclic voltammograms of solutions containing both cations at 296, 338, and 353 K are shown in Figure 5.

**Figure 5:**
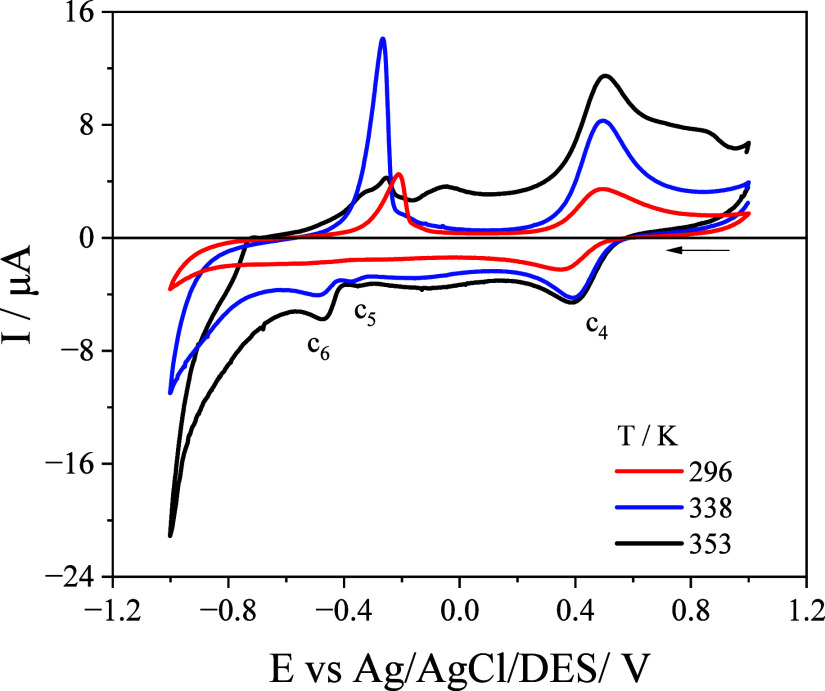
Cyclic voltammograms for 0.025 mol L^–1^ CuCl_2_.2H_2_O and 0.025 mol L^–1^ InCl_3_ in 1ChCl:2U on a platinum working electrode at 25 mV s^–1^ in different temperatures.

The reduction processes designated by c_4_, c_5_, and c_6_ were identified, as shown in Figure 5. The voltammograms for the cations at the evaluated temperatures are shown in Figure S5. For the solution containing only the Cu^2+^ species (Figure S3A), a reduction process was observed at 0.35 V. At the other temperatures, two processes, c”_4_ and c”_5_, were observed around −0.38 and −0.48 V, with only a change in current intensity. Figure S5B does not show reduction processes at 296 K; however, the c”_6_ process, at – 0.8 V and with a significant temperature dependence, was observed from changes in current: −6.3 and −11.0 μA at 338 and 353 K, respectively.

In Figure 5, the same reduction processes were observed with a significant shift in potential values. For the c_4_ process, the potential values remained unchanged from those shown in Figure S5A. The process called c_5_, however, occurred at approximately −0.36 V, a shift of 0.12 V compared to the absence of In^3+^. The c_6_ process at – 0.47 V was associated with the reduction of In^3+^/In^0^, with a change of 0.36 V, with the insertion of Cu^2+^ into the solution. Malaquias et al.^32^ also studied the CuIn alloy in 1ChCl:2U and obtained 0.5 V for Cu^2+^/Cu^+^ and 0.4 V for Cu^+^/Cu^0^, values that are relatively close to those obtained in this work. For In^3+^/In^0^, −0.6 V was observed, an intermediate value compared to those obtained in this research, considering that the experiments were carried out at 333 K. As shown in Figure 5, Malaquias et al.^32^ observed a significant change in the cyclic voltammograms of the solution containing Cu^2+^ and In^3+^ cations.

Analyses were performed using eq 1 to obtain the D values in the 1ChCl:2U medium. The data are organized in Table S2. The D_Cu_^2+^ values were 0.68, 1.48, and 4.03 × 10^–7^ cm^2^ s^–1^, while D_In_^3+^ presented the following results: 0.23, 1.13, and 1.38 × 10^–7^ cm^2^ s^–1^for 296, 338, and 353 K, respectively. For all temperatures, D_In_^3+^ showed lower values than those obtained for Cu^2+^, indicating a slower deposition kinetics. The relationship between the average D values was obtained for the species as a function of the inverse of viscosity, and, using eq 4, the r values for the evaluated species were obtained, as shown in Figure S6. It may be observed that the r values for Cu^2+^ increased up to 338 K and then decreased at 353 K; the opposite occurred for 1ChCl:2EG. For In^3+^ species, there was a linear increase with r values of 0.140 and 0.939 nm at 296 and 353 K, respectively. At 338 K, the r value was 0.532 nm, agreeing with the value obtained by Malaquias et al.:^32^ 0.6 nm for 333 K. The *E*_a_ values for Cu^2+^ and In^3+^ were 16.0 kJ mol^–1^ and 31 kJ mol^–1^, respectively. These values are consistent with the results obtained in Table S2, which indicate lower ionic mobility of In^3+^ (a smaller solvadynamic radius, diffusing a species more rapidly),^47^ confirmed by the reduced values of D and the size of the solvated radius, which indicates that In^3+^ is surrounded by molecules that end up requiring practically twice the energy compared to Cu^2+^.

### 3.6 SEM/XRD Analysis

CuIn coatings were prepared in 1ChCl:2U at 296 and 343 K on platinum substrates at different deposition potentials. The SEM results for coatings obtained at – 0.4, −0.8, and −1.0 V are shown in Figure 6. Figure 6 A and D shows results for the −0.4 V potential. The results showed a fine, homogeneous morphology at 296 K, similar to that reported by Cvetkovic et al.,^48^ and the presence of grains of different sizes scattered across the surface at 343 K. The EDS results indicated 100% Cu deposition, a finding confirmed by Figure 5, which shows that no In processes occur in this potential region.

**Figure 6:**
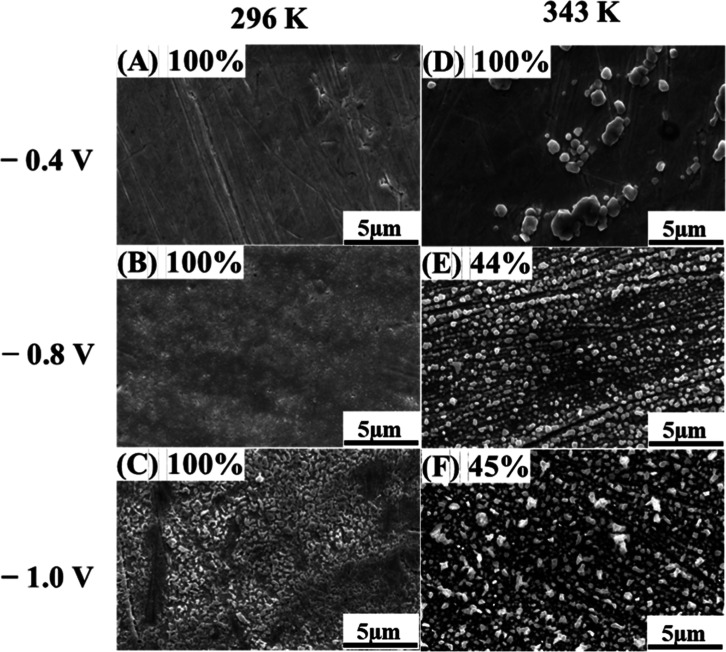
SEM images of CuIn electrodeposits over platinum for 0.025 mol L^–1^ CuCl_2_.2H_2_O + 0.025 mol L^–1^ InCl_3_ in 1ChCl:2U solution, applying different potentials for 3600 s at 296 and 343 K. By EDS, the Cu metallic composition was indicated in wt %.

By changing the potential to −0.8 V, a morphology similar to that observed at – 0.4 V at 296 K was obtained; however, at 343 K, 56% In was deposited, with a granular morphology showing various grain sizes across the surface. At – 1.0 V, the same predominance of Cu was observed as at the previous potentials (100% at 296 K), but the resulting morphology was not smooth, presenting small agglomerates across the surface, similar to that obtained by Malaquias et al.^32^ At 343 K, 55% In was received, but unlike the previous potential, it produced a morphology with larger grains and an overlayer, as reported in the literature for other deposition media.^30,38,48,49^

XRD analyses were performed on electrodeposited coatings using solutions containing 1:1 CuCl_2_ and InCl_3_ at potentials of −0.4, −0.8, and −1.0 V. Figure 7 shows the diffractograms of the electrodeposited study materials at 296 and 343 K.

**Figure 7:**
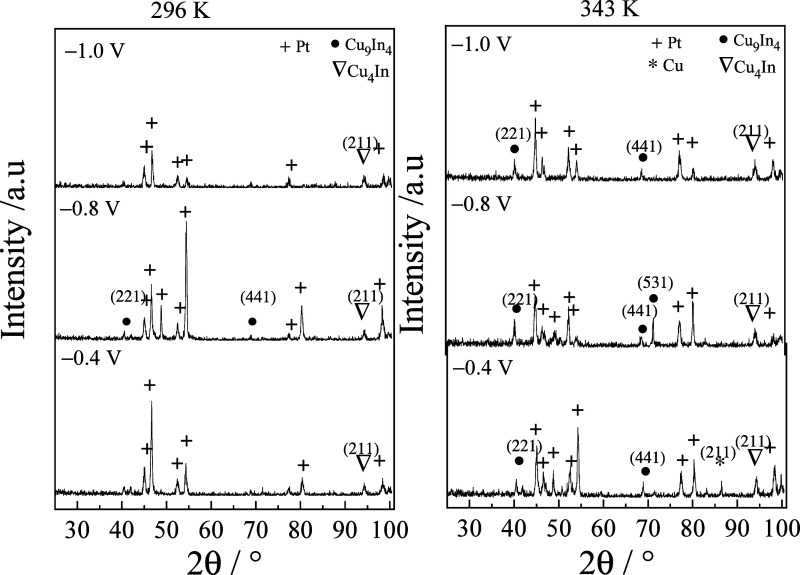
Electrodeposited Cu–In alloy diffractogram over the platinum surface for solution of 0.025 mol L^–1^ CuCl_2_ 2H_2_O + 0.025 mol L^–1^ InCl_3_ in 1ChCl:2U, applying the electrodeposition conditions shown in Figure 6 for 3600 s.

In all diffractograms in Figure 7, the peaks corresponding to the platinum substrate are indexed at 2θ = 45, 46.5, 52.5, 54.5, 77.5, 80, and 98.5°, with a cubic crystal structure (space group Fm3̅*m*, 225, ICDD 064921). Furthermore, a peak corresponding to the Cu_4_In phase (cubic spatial structure, space group Im3m, 229, PDF#00–042–1477), indexed at 94° (211), was identified in all materials electrodeposited at 296 and 343 K. The Cu_4_In alloy was not favored during electrodeposition processes, as other characteristic peaks of the phase were not identified at – 0.8 V; at 296 K, peaks corresponding to a Cu_9_In_4_ alloy (cubic spatial structure, space group *P*4̅3*m*, 215, PDF# 03–065–0703) were indexed at 40° (222) and 70° (441). In the electrodeposition processes at 296 K, in all samples, peaks referring to the Cu_9_In_4_ alloy phase were observed, indexed at 40° (222) and 70° (441), with an additional peak, indexed at 71.5° (531), in the diffractogram referring to the electrodeposition experiment at – 0.8 V, demonstrating that this experimental condition favored the formation of the Cu_9_In_4_ alloy.

Effect of the Molar Ratio in Coatings at 343 K.

The SEM results for CuIn coatings electrodeposited at – 0.8 and −1.0 V at 343 K with different Cu^2+^:In^3+^ ratios are shown in Figure 8. Considering a 4:1 Cu^2+^:In^3+^ ratio, 18% In deposition was observed at – 0.8 V and 22% at – 1.0 V. The morphology shown in Figure 8A was granular, with grains distributed across the surface, similar to the 1:1 morphology (Figure 6E). At – 1.0 V, the formation of islands of different sizes occurred, similar to that found in the literature above an overlayer^50^ identical to that obtained at – 0.8 V, where the ratio between the cations was approximately 4.5, while at the higher potential, the ratio was less than 3.6, indicating a good correlation with the proposed ratio. The 1:4 ratio showed a morphology like that reported by Sousa et al.^42^ for In-rich coatings. At – 0.8 V, the In content was 73%, while at – 1.0 V, it was 71%. This reduction in the measured values indicates that preferential deposition of In occurs at more positive potentials.

**Figure 8:**
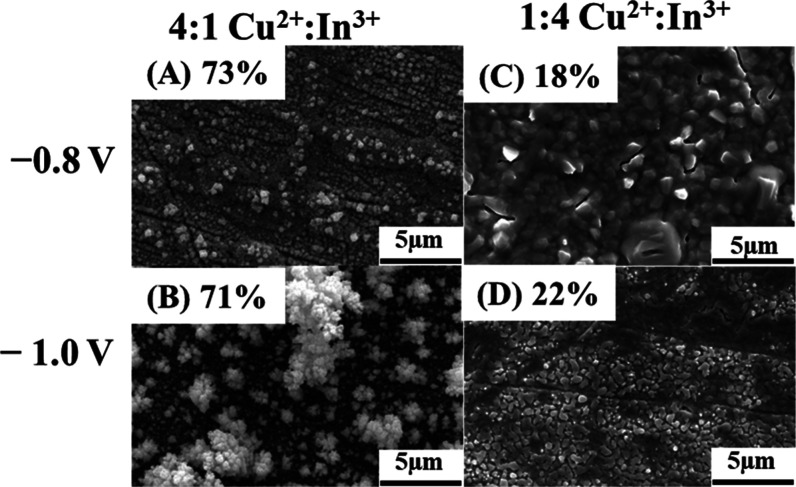
SEM images of CuIn electrodeposits on a platinum electrode in 1ChCl:2U solution at 343 K for different molar ratios. By EDS, the Cu metallic composition was indicated in wt %.

In Figures 6 and 8, the CuIn coatings obtained in the 1ChCl:2U solvent completely modified the grain type and size distribution, as seen in the SEM images at 296 K. An almost flat Cu surface was observed under this condition, even with increasing negative potentials. In this case, only with increasing temperature did a change in morphology occur due to the presence of indium after deposition at −0.8 V at 343 K. Since the grain sizes at −0.4 V are islands of pure copper, the deposition potentials also increased the In content and halved the electrodeposit grain size. This behavior is consistent with the electrochemical processes shown in Figure 5.

When coating solutions rich in In and Cu were heated to a relatively high temperature, the Cu-rich solution (Cu: In = 4:1) exhibited two distinct grain types, which directly influenced grain size. At – 0.8 V, the electrodeposits showed small aggregates on the main deposit, with relatively defined sizes, while the deposits at – 1.0 V, with inflorescence behavior due to the formation of cauliflower-like deposits, presented very different grain size and shape compared to the previous condition.

On the other hand, the indium-rich solutions (Cu: In = 1:4) apparently altered the indium content of the electrodeposited coating, although the grain size at – 0.8 V and −1.0 V did not show significant changes. Therefore, under this condition, the electrodeposition potential does not alter the morphology of the electrodeposit. Table 2 presents the ECD grain-size estimates for Figures 6 and 8.

**Table 2:** Grain Sizes Processed from SEM Images using the ECD Method

Cu/In (molar) ratio	temperature (K)	–*E*_DEP_ (V)	mean ECD ±sd (μm)
1:1	296	0.4	not detectable
		0.8	
		1.0	
	343	0.4	0.66 ± 0.18
		0.8	0.25 ± 0.25
		1.0	0.38 ± 0.34
4:1	343	0.8	0.75 ± 0.16
		1.0	1.78 ± 1.24
1:4	343	0.8	1.22 ± 0.54
		1.0	1.20 ± 1.22

## 4 Conclusions

Electrodeposition of CuIn films in 1ChCl:2EG and 1ChCl:2U media on a platinum electrode was successfully performed at different temperatures. The voltammograms showed reduction processes similar to those of the individual species, yielding 3 peaks in both solvents. For 1ChCl:2EG, the viscosity values for Cu^2+^ and In^3+^ in 1ChCl:2EG at 353 K were 7.9 and 8.1 mPa s, respectively. The r values for Cu^2+^ and In^3+^ varied from 0.408 to 0.325 nm and from 0.6 to 0.4 nm, respectively, with increasing temperature. The *E*_a_ for both species was approximately 34 kJ mol^–1^, and, considering that the difference in r values was not significant, the interaction of both species with the solvent was similar. For the 1ChCl:2U mixture, viscosity values were 21.0 and 20.0 mPa s at 353 K for Cu^2+^ and In^3+^, respectively. The r values increased proportionally with temperature, indicating that temperature increases affected the interactions of the species with the medium, with values of 0.3 nm for Cu^2+^ and 0.9 nm for In^3+^. The activation energy of In^3+^ was lower than that of Cu^2+^, with a value of 27.7 kJ mol^–1^. SEM analyses showed distinct morphologies at the analyzed temperatures in both solvents, with changes in grain size when the applied potential was varied during coating, allowing adjustment of the mass percentage of Cu or In depending on the desired application. XRD analyses revealed the presence of CuIn and Cu_2_In phases in 1ChCl:2EG and Cu_9_In_4_ and Cu_4_In phases in 1ChCl:2U, indicating that, depending on the solvent used, different CuIn phases can be obtained, which may have various applications.

In 1ChCl:2EG, the estimated grain size doubled and increased approximately with increasing temperature. However, in both ratios (1:1 and 1:2), the grains did not change in size when the temperature was changed from 338 to 353 K (0.34 and 0.60 μm for 1:1 and 1:2, respectively). In 1ChCl:2U, changing the ratio altered the grain size at – 0.8 V approximately three times (0.75 ± 0.16 μm), and the largest value was obtained at – 1.0 V in the 4:1 ratio (1.78 ± 1.24 μm).

## Supplementary Material



## Notes

Analyses of cyclic voltammetry, Stokes–Einstein plots, particle size distribution and specific mass, dynamic viscosity, and diffusion coefficient (PDF)
