# Supplementary material for: Sustainable CuIn Electrodeposition from Deep Eutectic Solvents: Influence of Ethylene Glycol and Urea
Source: ACS Omega. 2026 Jul 31;11(31):45939–48. doi: 10.1021/acsomega.6c02001 (PMC13470812; doi:10.1021/acsomega.6c02001)
Supplement: Supplementary file 1 [file ao6c02001_si_001.pdf]

Supplementary Material

Sustainable CuIn electrodeposition from deep eutectic solvents: Influence of ethylene glycol and urea

David A. de Assis[a#], José W. A. Crisóstomo[a#], Ana A. C. Alcanfor[a], Othon S. Campos[b], Luciana Vieira[c], Filipe X. Feitosa[d], Hosiberto B. de Sant'Ana[d], Diego F. Dias[e], Edipo S. de Oliveira[f], Hamilton F.G. de Abreu[f], Natalia G. Sousa[a,d], Francisco M. T. de Luna[d], Pedro de Lima-Neto[a], Adriana N. Correia[a]*

[a]Departamento de Química Analítica e Físico-Química, Centro de Ciências, Universidade Federal do Ceará, Campus do Pici, Bl 940, 60440-900, Fortaleza, Ceará, Brazil

[b]Departamento de Química e Física, Centro de Ciências Exatas, Naturais e da Saúde, Universidade Federal do Espírito Santo, Campus Alegre, 29500-000, Alegre, Espírito Santo, Brazil

[c]Fraunhofer Institute for Interfacial Engineering and Biotechnology IGB Bio-, Electro- and Chemocatalysis BioCat, Straubing Branch, Schulgasse 11a, Straubing 94315, Germany

[d]Departamento de Engenharia Química, Centro de Tecnologia, Universidade Federal do Ceará, Campus do Pici, Bl 709, 60440-900, Fortaleza, Ceará, Brazil

[e]Programa de Pós-Graduação em Física, Centro de Ciências, Universidade Federal do Ceará, Campus do Pici,60440-900, Fortaleza, Ceará, Brazil

[f]Departamento de Engenharia Metalúrgica e de Materiais, Universidade Federal do Ceará, Campus do Pici, 60440-900, Fortaleza, Ceará, Brasil

KEYWORDS: Electrodeposition; chronoamperometry; diffusion coefficients; ethaline; reline.

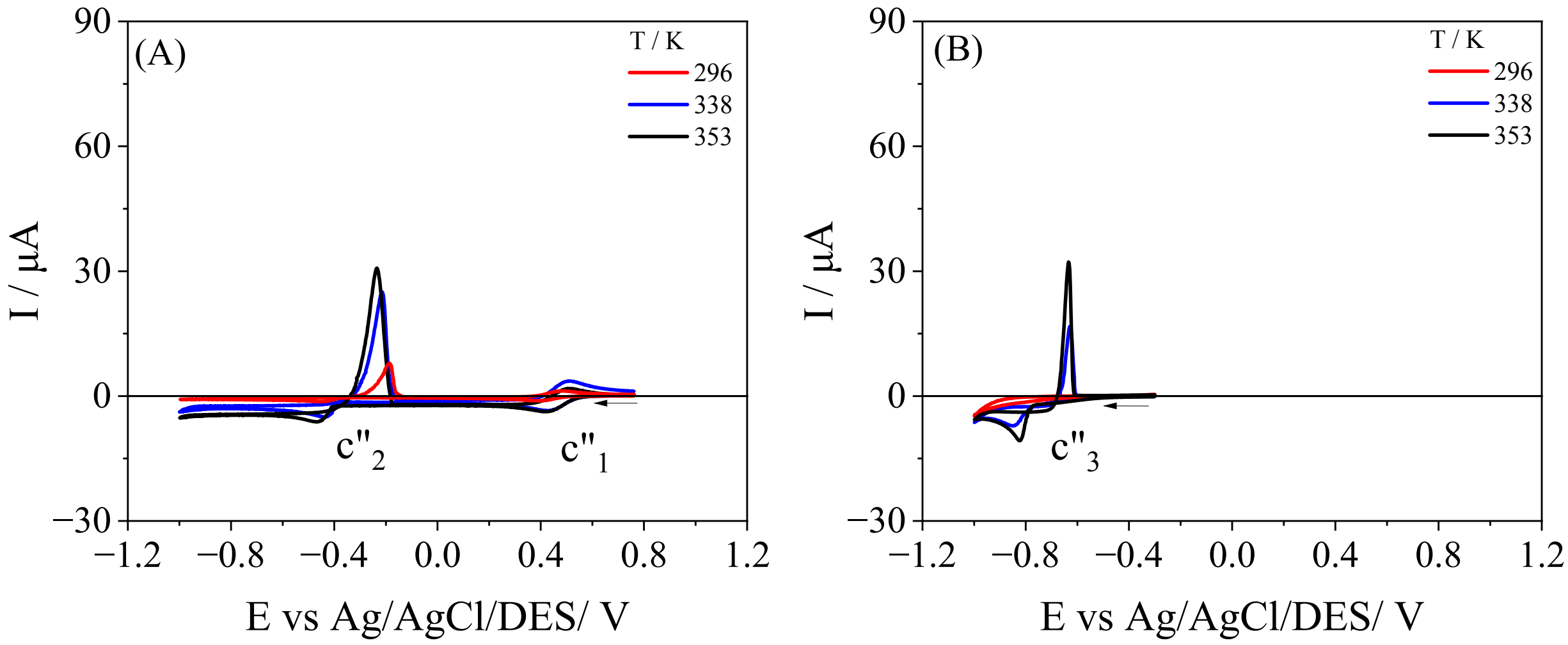

Figure. S1. Cyclic voltammograms for 0.050 mol L^{-1} $CuCl_2.2H_2O$ (A) and 0.050 mol L^{-1} $InCl_3$ (B) in 1ChCl:2EG on platinum working electrode at 25 mV s^{-1} in different temperatures.

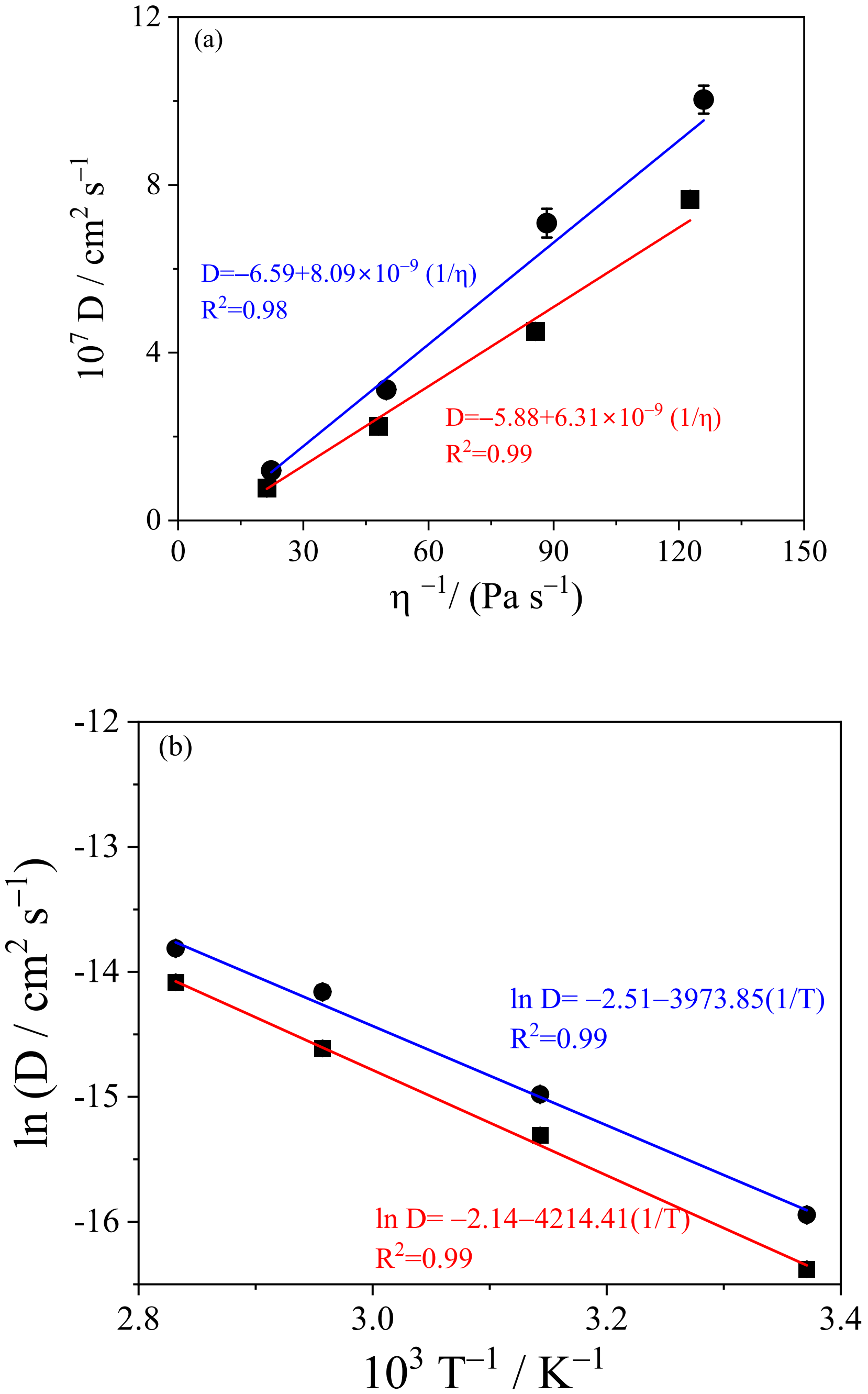

Figure. S2. (a) Stokes-Einstein's plot for 1ChCl:2EG containing 0.05 mol L^{-1} $CuCl_2$. (circle) and 0.05 mol L^{-1} $InCl_3$ (square) solutions in function of viscosity. (b) Arrhenius' plot for 0.05 mol L^{-1} $CuCl_2.2H_2O$ (circle), 0.05 mol L^{-1} $InCl_3$ (square) solutions in function of temperature.

Grain counting methodology

The SEM images obtained were subjected to granulometric analysis by pixel conversion using the image scale bar. To obtain the most reliable grain distribution possible from these images, the equivalent circular diameter (ECD) method proposed by Jennings and Parslow[1] for irregular particles was used. The ECD value was considered using the following equation:

$$d_{ECD} = 2\sqrt{\frac{A_{\mu m^2}}{\pi}} \qquad \text{S1}$$

Where is d_{ECD} the effective ECD from this model and $A_{\mu m}^2$ the projected particle area from the SEM figures using this method. Those areas were obtained by using a calibration factor using the following equation:

$$k = \frac{5\times10^{-6}}{L_{scale}} \qquad \text{S2}$$

Where L_{scale} represents the length of the scale in pixels and k represents the scale factor in m px^{-1}. Having taken the length, the area was calculated using the equation below:

$$A_{\mu m^2} = A_{px} \times k^2 \qquad \text{S3}$$

Where $A_{\mu m^2}$ refers to the projected area shown in Equation S1, A_{px} is the pixelized area from SEM images in px^2. For a precise calculation of d_{ECD}, a Python script was written in order to increase the precision and automatize the methodology. Since then, the OpenCV[2], scikit-image[3], SciPy[4] and NumPy[5] packages were used in this script. For obtaining the distribution data for having the diameter histogram, the following equation was applied:

$$\langle d \rangle = \max(v,h) \times k \qquad \text{S4}$$

Where $\langle d \rangle$ is the counting of the particles in the SEM image, and $\max(v,h)$ refers to the maximum values of, respectively, pixelized vertical and horizontal extensions of the particles in SEM images,

and k has the same meaning shown in Eq. S2. Within the script, the original SEM images were copied and treated in order to optimize this calculation and obtain the grain distribution among those data.

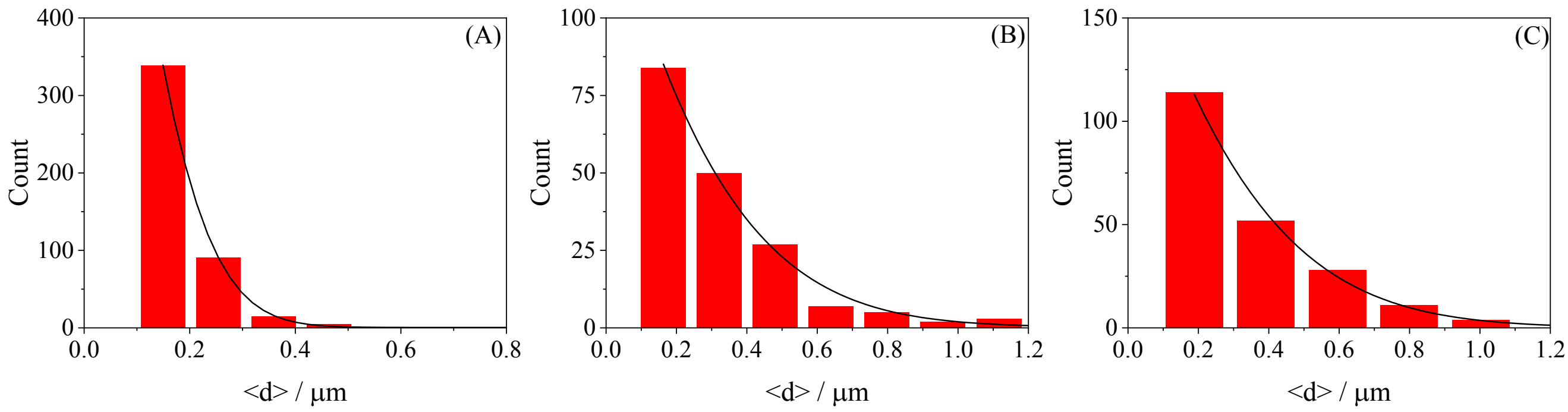

Figure S3. Distribution of the CuIn grain distribution from Figure 2 using ECD method

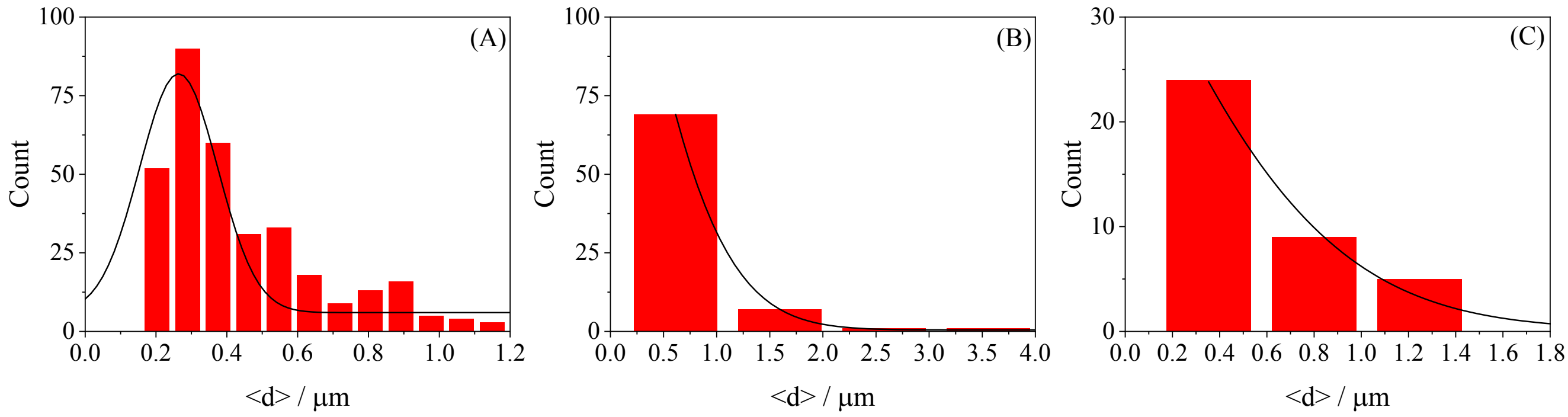

Figure S4. Distribution of the CuIn grain distribution from Figure 4 using ECD method

The Figures S3 and S4 show the particle distribution obtained by SEM images from Figures 2 and 4. Except for Figure S3A, which presented a Gaussian fitting, other figures presented a half (or less than half) Gaussian fitting, and the particle histograms were biased from the left (or lower particle sizes). Considering the low particle counts and the size behaviour in SEM imaging, such as particle aggregation and the limitations of the ECD method, which does not measure correctly fused grains, the histograms showed a different distribution, albeit a tendency was observed.

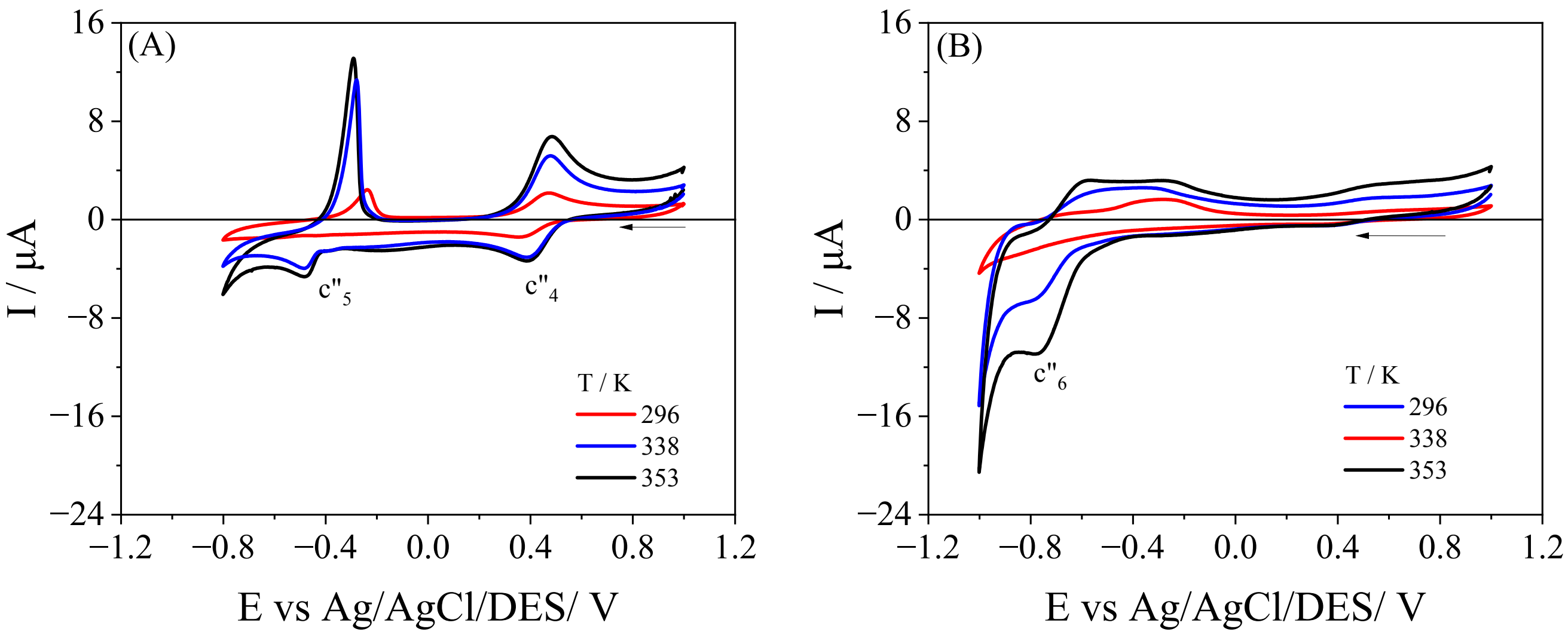

Figure. S5. Cyclic voltammograms for 0.025 mol L^{-1} $CuCl_2$ (A) and 0.025 mol L^{-1} $InCl_3$ (B) in 1ChCl:2U on platinum working electrode at 25 mV s^{-1} in different temperatures.

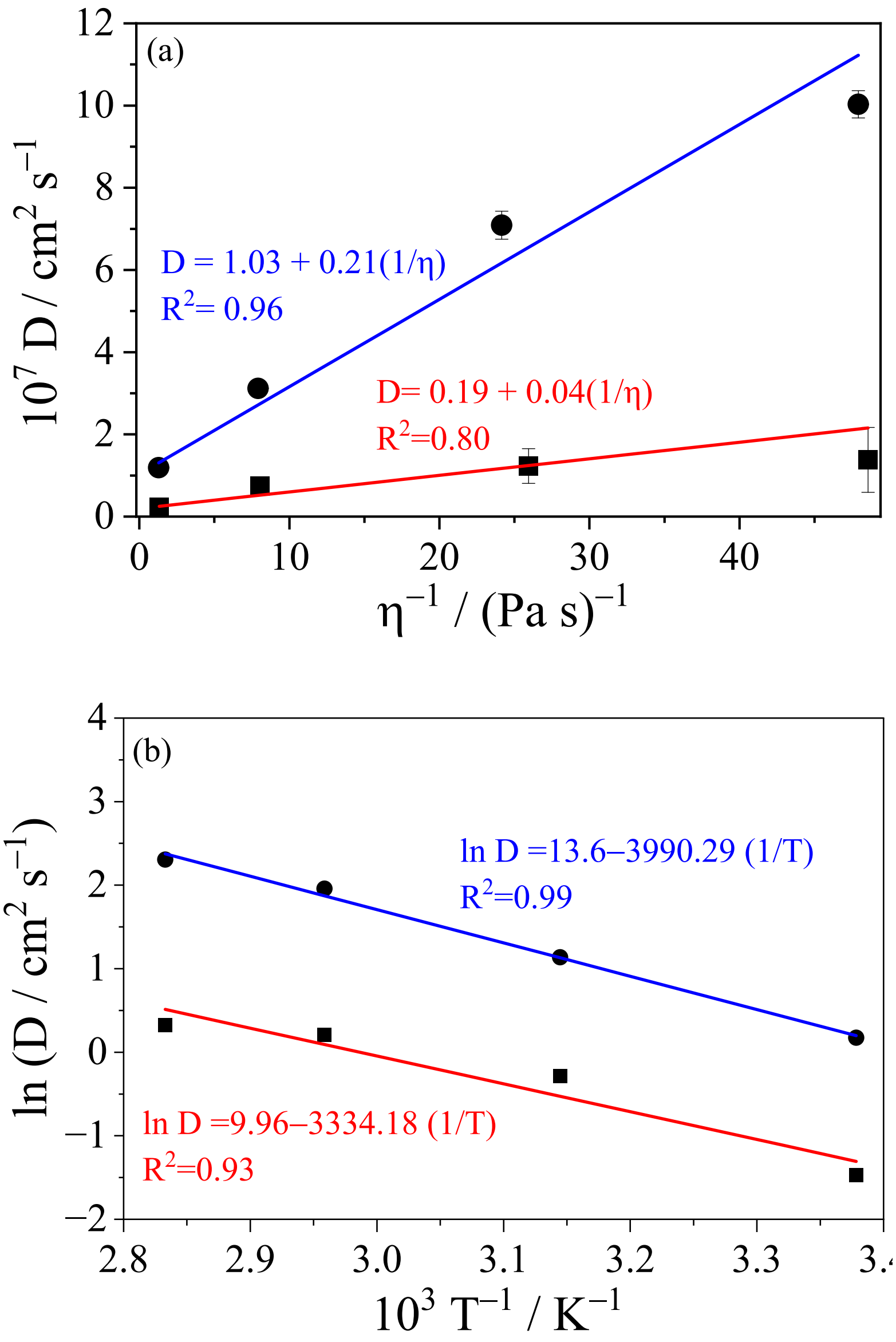

Figure S6. (a) Stokes-Einstein's plot for 1ChCl:2U containing 0.025 mol L^{-1} $CuCl_2$. (circle) and 0.025 mol L^{-1} $InCl_3$ (square) solutions in function of viscosity. (b) Arrhenius' plot for 0.025 mol L^{-1} $CuCl_2.2H_2O$ (circle), 0.0250 mol L^{-1} $InCl_3$ (square) solutions in function of temperature.

Table S1. Specific mass (ρ), dynamic viscosity (η), and diffusion coefficient (D), solvodynamic radius (r) and apparent activation energy and water content of 1ChCl:2EG solutions containing metallic ions in different temperatures.

**Solutions**	**T (K)**	ρ **(g cm⁻³)**	η **(mPa s)**	D **(10⁻⁷ cm² s⁻¹)**	$r_{Cu^{2+}}$ **(nm)**	$E_{Cu^{2+}}$ **(kJ mol⁻¹)**
0.05 mol L^{-1} $CuCl_2$	296	1.1214	44.816	1.19 ± 0.05	0.408	33.52
	318	1.1090	20.045	3.12 ± 0.09	0.373	
	338	1.0978	11.318	7.09 ± 0.34	0.309	
	353	1.0894	7.9395	10.03 ± 0.33	0.325	
**Solutions**	**T (K)**	ρ **(g cm⁻³)**	η **(mPa s)**	D **(10⁻⁷ cm² s⁻¹)**	$r_{Cu^{2+}}$ **(nm)**	$E_{Cu^{2+}}$ **(kJ mol⁻¹)**
0.05 mol L^{-1} $InCl_3$	296	1.1243	47.042	0.77 ± 0.01	0.600	35.10
	318	1.1117	20.823	2.25 ± 0.04	0.498	
	338	1.1006	11.680	4.51 ± 0.06	0.471	
	353	1.0921	8.1514	7.66 ± 0.07	0.415	
**Water content**						
1ChCl:2EG	29.5± 0.40					
CuIn EG1:1	20.5±1.11					

Table S2. Specific mass (ρ), dynamic viscosity (η), and diffusion coefficient (D), solvodynamic radius (r), activation energy and water content of 1ChCl:2U solutions containing metallic ions in different temperatures.

**Solutions**	**T (K)**	**ρ (g cm^{-3})**	**η (mPa s)**	**D (10^{-7} cm^2 s^{-1})**	**$r_{Cu^{2+}}$ (nm)**	**$E_{Cu^{2+}}$ (kJ mol^{-1})**
0.025 mol L^{-1} $CuCl_2$	296	1.1969	791.86	0.68 ± 0.17	0.041	33.15
	318	1.1847	130.25	1.17 ± 0.20	0.153	
	338	1.1735	42.307	1.48 ± 0.10	0.395	
	353	1.1646	21.017	4.03 ± 1.92	0.305	
**Solutions**	**T (K)**	**ρ (g cm^{-3})**	**η (mPa s)**	**D (10^{-7} cm^2 s^{-1})**	**$r_{Cu^{2+}}$ (nm)**	**$E_{Cu^{2+}}$ (kJ mol^{-1})**
0.025 mol L^{-1} $InCl_3$	296	1.1965	681.03	0.23 ± 0.05	0.140	27.7
	318	1.1840	121.16	0.75 ± 0.16	0.257	
	338	1.1729	37.970	1.23 ± 0.42	0.532	
	353	1.1642	20.013	1.38 ± 0.79	0.939	
**Water content**						
		1ChCl:2U	22.2±0.21			
		CuIn 1:1	30.6±0.61			
		CuIn 1:4	30.3±1.11			
		CuIn 4:1	31.7±0.60			

References

(1) B. R. Jennings,K. Parslow; Particle size measurement: the equivalent spherical diameter. Proc. A1 September 1988; 419, 137–149.

(2) G. Bradski, "The OpenCV Library," *Dr. Dobb's Journal of Software Tools*, **2000,** 25, 120–125.

(3) Van der Walt, S. J., Schönberger, J. L., Nunez-Iglesias, J., Boulogne, F., Warner, J. D., Yager, N., Gouillart, E., Yu, T., & the scikit-image contributors. scikit-image: image processing in Python. PeerJ, **2014**, *2*, e453.

(4) Virtanen, P., et al. SciPy 1.0: Fundamental algorithms for scientific computing in Python. Nature Methods, **2020,** 17(3), 261-272.

(5) Harris, C.R., et al. "Array programming with NumPy." Nature. **2020,** 585, 357–362.
